# Fiber-based lactate recordings with fluorescence resonance energy transfer sensors by applying an magnetic resonance-informed correction of hemodynamic artifacts

**DOI:** 10.1117/1.NPh.9.3.032212

**Published:** 2022-05-09

**Authors:** Henriette Lambers, Lydia Wachsmuth, Dominik Thomas, Fawzi Boumezbeur, Vanessa Hoesker, Bruno Pradier, Cornelius Faber

**Affiliations:** a University Hospital Münster, Translational Research Imaging Center (TRIC), Clinic for Radiology, Münster, Germany; b NeuroSpin, CEA, CNRS, Paris-Saclay University, Gif-Sur-Yvette, France

**Keywords:** multimodal fMRI, fluorescence, fiber-based, fluorescence resonance energy transfer, blood oxygenation level-dependent, CBV

## Abstract

**Significance:**

Fluorescence resonance energy transfer (FRET) sensors offer enormous benefits when studying neurophysiology through confocal microscopy. Yet, their use for fiber-based *in vivo* recordings is hampered by massive confounding effects and has therefore been scarcely reported.

**Aim:**

We aim to investigate whether *in vivo* fiber-based lactate recordings in the rodent brain are feasible with FRET sensors and implement a correction algorithm for the predominant hemodynamic artifact.

**Approach:**

We performed fiber-based FRET recordings of lactate (Laconic) and calcium (Twitch-2B) simultaneously with functional MRI and pharmacological MRI. MR-derived parameters were applied to correct hemodynamic artifacts. Results of FRET measurements were validated by local field potential, magnetic resonance spectroscopy, and blood analysis.

**Results:**

Hemodynamic artifacts dominated fiber-based *in vivo* FRET measurements with both Laconic and Twitch-2B. Our MR-based correction algorithm enabled to remove the artifacts and detect lactate and calcium changes during sensory stimulation or intravenous lactate injections.

**Conclusions:**

*In vivo* fiber-based lactate recordings are feasible using FRET-based sensors. However, signal corrections are required. MR-derived hemodynamic parameters can successfully be applied for artifact correction.

## 1 Introduction

Lactate has long been merely considered a waste product of anaerobic metabolism that, however, can be utilized to indicate pathologic conditions, such as cancer^1^^,^^2^ or inflammatory and autoimmune diseases.^3^^,^^4^ Evidence is growing that brain lactate plays important role in energy metabolism, maintaining homeostatic functions, and acting as a signaling molecule. Investigating the underlying mechanisms is a matter of current research.^5^^,^^6^ *In vivo*, cerebral lactate measurements remain challenging since physiologic lactate levels are low compared with other metabolites.^7^ Cerebral lactate can be detected by using magnetic resonance spectroscopy (MRS),^8^^–^^10^ microdialysis,^11^^–^^13^ or cyclic voltammetry.^14^^,^^15^ However, only a bulk signal or extracellular lactate can be measured using these methods. In addition, for MRS and microdialysis, only a low temporal resolution is achievable. These shortcomings particularly impede the investigation of cerebral energy metabolism. Resolving the distinct roles of neurons and astrocytes requires cell-specific detection of lactate levels with high temporal resolution.^16^^,^^17^ A genetically encoded lactate sensor, Laconic,^18^ has recently been developed and was successfully used for cell-specific *in vivo* lactate recordings in mice using two-photon microscopy.^19^^,^^20^

Combining functional magnetic resonance imaging (fMRI) and fluorescence recordings of genetically encoded lactate sensors has the potential to provide insight into cerebral lactate metabolism by complementing local lactate levels with a whole-brain view of hemodynamic activity. Although magnetic resonance (MR) compatible microscopes have been developed^21^^,^^22^ and cortex-wide calcium imaging has been successfully performed during fMRI,^23^ fiber-based fluorescence recordings^24^ have found widespread application for simultaneous fluorescence-fMRI recordings.^25^^–^^34^

Laconic is a fluorescence resonance energy transfer (FRET) sensor. Generally, FRET metabolic sensors consist of a sensing domain and two fluorophores, referred to as donor and acceptor, which are capable of FRET from the blue-shifted donor to the red-shifted acceptor.^35^ The sensor signal is obtained as the ratio of light emitted by each of the fluorophores. In contrast to single-wavelength fluorescence sensors, FRET requires the detection of light from both fluorophores at different wavelengths. Natsubori et al.^36^ successfully performed *in vivo* fiber-based fluorescence measurements in animals expressing a FRET-based calcium sensor. Yet, potential confounders for fiber-based FRET recordings such as altered cerebral blood flow or oxygenation changes have not been investigated in detail, as compared with reports for both fiber-based fluorescence measurements^33^ and microscopy^23^^,^^37^^,^^38^ using single-wavelength detection. For fiber-based lactate detection, it appears to be of particular importance to correct for such potential artifacts due to the low cerebral lactate level.

In this study, we investigated whether *in vivo* fiber-based FRET recordings enable the detection of changes in lactate level. We first instituted a FRET-based calcium sensor, Twitch-2B,^39^ with a known impulse response function (IRF) for investigating the hemodynamic artifact. As MR contrast is intrinsically sensitive to hemodynamic changes,^40^ we then used MR-derived parameters to correct hemodynamic artifacts in fiber-based fluorescence recordings.

## 2 Materials and Methods

Animal experiments were carried out according to the German Animal Welfare Act and were approved by the State Agency for Nature, Environment and Consumer Protection of North Rhine-Westphalia, Germany (LANUV approval IDs 84-02.04.2015.A427, 84-02.04.2016.A135, and 81-02.04.2018.A426). Experiments were performed on 43 adult female Fischer rats [>3 months, (183±12)  g]. Rats were housed in groups of two to five animals under a regular light/dark schedule (12/12 h) with food and water ad libitum.

**Fig. 1:**
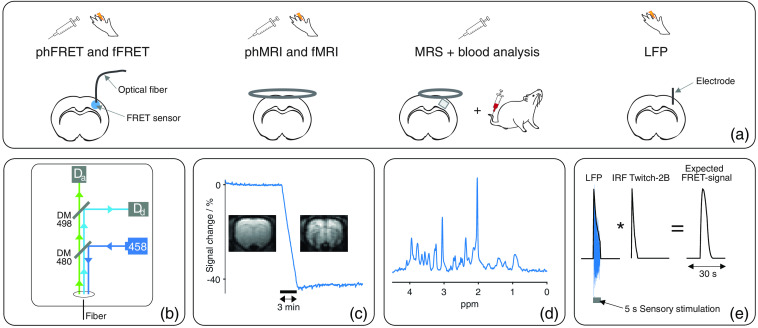
(a) Study design: functional (f) and pharmacological (ph) experiments were performed using fiber-based FRET recordings, MRI, MRS, blood analysis, or LFP recordings. For FRET recordings, a fiber was implanted in the S1Fl of Twitch-2B- or Laconic-expressing animals. For MRI and MRS, a 2- and 1-cm surface coil were used, respectively. A $9\;{\mathrm{mm}}^{3}$ cortical voxel was investigated using MRS. For blood analysis, blood was sampled from the tail vein. LFP signals were recorded as difference between the electric currents of S1Fl and cerebellum using implanted electrodes. (b) Schematic of fiber-based FRET setup: a laser (458 nm) emitted excitation light (dark blue). Excitation light was reflected by a dichroic mirror (CWL: 480 nm) and coupled into a fiber. Fluorescent light emitted by the FRET sensors was separated using a second dichroic mirror (CWL: 498 nm): Fluorescent light emitted by donor fluorophores (light blue) was reflected and detected by an APD ($D_{d}$). Light emitted from acceptor fluorophores (green) transmitted to the mirror and was detected by a second APD ($D_{a}$). (c) Exemplary MRI measurement during contrast agent injection: signal decreases roughly 40% altering contrast notably. (d) Exemplary *in vivo* H1 NMR spectrum. (e) Calculation of expected FRET ratios of Twitch-2B-expressing animals: Envelope of LFP response (blue) to sensory stimulation was convolved with the IRF of Twitch-2B.^39^

This study consisted of fiber-based optical recordings in animals expressing the genetically encoded FRET-based calcium (Twitch-2B) or lactate (Laconic) sensor in the sensory cortex, fMRI, and pharmacological MRI (phMRI) to understand and correct hemodynamic artifacts and local field potential (LFP) recordings, MRS measurements as well as blood analysis for interpretation of FRET signals [Fig. 1(a)].

We performed fiber-based *in vivo* functional FRET (fFRET) fluorescence measurements simultaneously with fMRI (fMRI-FRET) upon short sensory stimulation. Additionally, pharmacological FRET (phFRET) was conducted in two separate experiments: first, contrast-agent-free EPI measurements (phEPI) were performed simultaneously with phFRET (phEPI-FRET). Second, pharmacological cerebral blood volume-weighted (CBV) MR measurements were conducted (phCBV). We estimated the expected fFRET signal for Twitch-2B expressing animals using LFP measurements. For interpretation of phFRET measurements, we examined whether the injected metabolite penetrates the blood-brain barrier using blood analysis and MRS measurements. An overview of all individual experiments is shown in Table S1 in the Supplemental Materials.

### 2.1 Animal Handling

Animal preparation and surgical procedures were performed under isoflurane anesthesia (5% induction, 2% to 3% maintenance, in 1  L/min $O_{2}$). Blood sampling was performed under 1.8% isoflurane anesthesia. All other experiments were performed under medetomidine sedation, which was induced by a subcutaneous bolus injection of 0.04  mg/kg and followed by a continuous infusion of 0.05  mg/kg/h. After medetomidine bolus, isoflurane was discontinued within 10 to 15 min and experiments were executed at least 40 min after bolus injection. Medetomidine sedation was supplemented by 0.4% isoflurane in eight pharmacological experiments. This attenuated the increase in respiration rate due to lactate infusion (Table S1 in the Supplemental Materials).

About 30 min prior to stereotaxic surgery for virus injection, fiber or electrode implantation, the animals received analgesia [Metacam (1  mg/kg s.c.) or Metamizol (100  mg/kg s.c.)].

16 animals were ventilated (MRI-1 Ventilator, CWE, Inc., Ardmore, Pennsylvania, United States). Three of them received the muscle relaxant Pancuronium (2  mg/kg bolus followed by continuous injection of 1.5  mg/kg/h). The end-expiratory ${\mathrm{CO}}_{2}$ was continuously monitored in ventilated animals using a ${\mathrm{CO}}_{2}$ analyzer (Micro CapStar End-Tidal ${\mathrm{CO}}_{2}$ Analyzer, CWE, Inc., Ardmore, Pennsylvania, United States). Respiration rate of ventilated animals was set to 53 breaths per minute (bpm). The respiratory minute volume was between 95 and 127  mL/min. Respiration rate of spontaneously breathing animals was (60±15)  bpm. Animals were placed on a temperature-controlled bed and fixated with bite and ear bars. Rectal temperature was held at (36.6±0.3)°C.

### 2.2 Functional and Pharmacological Measurements

For functional measurements, electrical paw stimulation was used. For this purpose, electrodes were inserted into the forepaw contralateral to the implanted fiber/electrode. 1 ms pulses were applied at a frequency of 9 Hz and an amplitude of 1.5 mA according to a block paradigm (5-s stimulation, 25-s rest, and six repetitions). For LFP, recordings of one stimulation period were sufficient.

For pharmacological measurements, a 500 mM solution of l-lactate was injected via a lateral tail vein catheter. For phFRET, phMRI and MRS 2.5 or 2.6  mmol/kg lactate were injected over 180 s (Table S1 in the Supplemental Materials). For blood analysis, a lower lactate concentration (250 mM) was used and 1.0  mmol/kg lactate was injected in 150 s. This ensured that the maximum detectable lactate level of the detection device (Sec. 2.6) was not exceeded. As control, phFRET and phMRI measurements were performed by injecting 5  mL/kg saline over 180 s.

### 2.3 Fiber-Based FRET Fluorescence Recordings

#### 2.3.1 Animal preparation for fiber-based fluorescence recordings

Preparation procedures for fiber-based fluorescence recordings have been described in detail previously.^41^ AAVs encoding Twitch-2B or Laconic were injected into forelimb region of primary somatosensory cortex (S1Fl) at least 4 weeks prior to fluorescence recordings. Histology confirmed that FRET sensors were mainly expressed in neurons. Detailed information about the viral vectors is given in the supplement (Fig. S1 and Table S2 in the Supplemental Materials). Immediately before the fluorescence experiment, an optical fiber (400-μm diameter) was implanted ∼100  μm above the sensor expressing region and glued to the skull.

Laconic/pcDNA3.1(-) was a gift from Luis Felipe Barros (Addgene plasmid # 44238;^42^ RRID:Addgene_44238). pAAV.hSyn1.Twitch-2B.WPRE.SV40 was a gift from Oliver Griesbeck (Addgene plasmid # 100040;^43^ RRID:Addgene_100040).

#### 2.3.2 Procedure of fiber-based fluorescence recordings

A custom-built setup was used for fiber-based fluorescence recordings [Fig. 1(b)]. This setup was similar to setups commonly used for fiber-based fluorescence recordings, which mainly consist of a light source, a dichroic mirror, and a light detector. To enable FRET-based fluorescence recordings a second pair of dichroic mirror and light detector was added to detect the light emitted from donor and acceptor fluorophores separately.

The excitation and emission spectra of the fluorophores of Twitch-2B and Laconic (Figs. S2(a) and S2(b) in the Supplemental Materials) resulted in two specific requirements for the optical setup. First, the excitation light wavelength should be near the maximum of the excitation spectrum of donor fluorophores (around 450 nm^44^^,^^45^). Second, a dichroic mirror was required to separate blue light (around 480 nm^44^^,^^45^) emitted by the donor fluorophores from green light emitted by acceptor fluorophores (around 530 nm^46^).

In detail, the setup was composed as follows [Fig. S2(c) and Table S3 in the Supplemental Materials]. Excitation light was supplied by a blue laser (458 nm), reduced by neutral density filters, and reflected by a dichroic mirror [central wavelength (CWL): 480 nm]. A collimator focused the excitation light into the implanted optical fiber and the sensor expressing region was constantly illuminated via the fiber (25 to 160  μW at the fiber tip). Fluorescence light emitted by the donor and acceptor fluorophores was collected by the fiber, passed the dichroic mirror, a notch filter (CWL: 457 nm), and was separated by a second dichroic mirror (CWL: 498 nm). The light emitted by donor fluorophores was reflected by the mirror and went through a bandpass filter (CWL: 479 nm), whereas the light emitted by acceptor fluorophores was transmitted to the mirror and passed a longpass filter (cut-on wavelength: 480 nm). The separated fluorescence light was focused by two lenses and detected by two avalanche photodiodes (APDs). Fluorescence signals were recorded at 2 kHz using a multifunction data acquisition device (PCIe-6363, National Instruments, Austin, Texas, United States) and a custom-written LabView script (National Instruments).

It should be noted that a portion of the excitation light was reflected at the fiber instead of being collimated. Although the used notch filter removed most of this reflected light, some light fell onto the detectors. The resulting technical background noise was calibrated for each laser light intensity and was subtracted from the detected fluorescence signals.

### 2.4 MR Measurements

All MR measurements were acquired on a 9.4 T Bruker Biospec 94/20 small animal scanner equipped with a 720  mT/m gradient system (Bruker BioSpin GmbH, Ettlingen, Germany), a linearly polarized resonator, and a receive-only surface coil with 2 cm (MRI) or 1 cm (MRS) diameter. First, an anatomical image was acquired to identify the slice position for echo-planar imaging (EPI) or the voxel position for spectroscopy: 2D Turbo spin echo sequence (RARE), $\mathrm{TR}/{\mathrm{TE}}_{\mathrm{Eff}}=2000/50\;\mathrm{ms}$, RARE factor 8, Matrix 256×256, field of view (FOV) $28\times 26\;{\mathrm{mm}}^{2}$, slice thickness 1.2 mm, nine contiguous slices. Subsequently, $B_{0}$ homogenization in the area of interest was achieved using the MAPSHIM Bruker routine.

#### 2.4.1 GE-EPI and CBV measurements

Gradient-echo-EPI (GE-EPI) and CBV measurements were performed using a single-shot gradient-echo EPI sequence (TR 1 s, same FOV as anatomy; GE-EPI: TE 18.0 ms, Matrix 80×80, flip angle 60 deg, nine slices; CBV: TE 8.2 ms, Matrix 64×64, flip angle 52 deg, three slices). Measurements lasted 3 min for functional measurements and 5 min for pharmacological measurements (2-min baseline, 3-min injection).

For CBV measurements the contrast agent Molday Ion (30-nm particle size, BioPAL, Worcester, Massachusetts, United States), containing superparamagnetic iron oxide nanoparticles, was injected into the catheterized tail veins of nine animals (15 mg Fe/kg). Injections were performed during 25 min lasting CBV-EPI measurement [10-min baseline, 3-min injection, 12-min baseline; Fig. 1(c)]. Subsequently, a pharmacological measurement (phCBV) was performed. Four animals received the lactate solution, and five animals were given saline injections.

#### 2.4.2 H1 MRS

Localized H1 spectra were acquired from a cortical volume-of-interest ($\mathrm{VOI}=3\times 1\times 3\;{\mathrm{mm}}^{3}$) using a LASER sequence^47^ [TE/TR=27.2/2000  ms, 2048 points, VAPOR water suppression,^48^ 90 or 150 averages; Fig. 1(d)]. A chemical shift displacement artifact of 15% was expected between the water and lipid signals. Water linewidths were (13.2±1.4)  Hz after $B_{0}$ homogenization. Series of H1 spectra were performed before (up to five), during (one or two spectra), and after the intravenous infusion of the lactate solution.

### 2.5 LFP Measurements

For LFP recordings, two custom-made silver electrodes (250-μm diameter) were implanted in the brain of 10 animals. One electrode was placed in the S1Fl and the reference electrode was positioned in the cerebellum. Electrodes were glued to the skull using dental cement. LFP signals were recorded as the difference between the electric currents detected by the electrodes using a differential amplifier (DPA-2FX, NPI Electronics, Tamm, Germany) with an implemented amplifier and bandpass filter (HP: 3 Hz and LP: 30 Hz). Signals were acquired at 2 kHz using the same data acquisition device and LabView script as for the fluorescence recordings.

### 2.6 Blood Analysis

Blood lactate levels were obtained in three animals before and after infusion of the l-lactate solution under isoflurane anesthesia. 100-μL blood was sampled from the lateral tail vein, using heparinized glass capillaries and analyzed using a blood analyzer (Vet Scan i-STAT1, Abaxis, Union City, California, United States). Under medetomidine sedation, blood sampling was omitted due to peripheral vasoconstriction.

### 2.7 Data Preprocessing

Analysis was done using MATLAB (Release 2018b, The MathWorks, Inc., Natick, Massachusetts, United States) if not explicitly specified otherwise. EPI datasets of GE EPI and CBV measurements were realigned using SPM 12 (Statistical Parametric Mapping, Functional Imaging Laboratory, Wellcome Trust Centre for Neuroimaging, London, United Kingdom). The first five scans of each dataset were discarded to avoid presteady-state artifacts. In each dataset, a region of interest (ROI) was positioned in S1FL with the implanted fiber within the ROI.

For fMRI blood oxygenation level-dependent (BOLD) data a voxelwise Mann–Whitney U-test^49^ was performed within the chosen ROI to find significantly activated voxels. The U-test examined whether the signals of rest and stimulation periods differed significantly after Bonferroni correction (p<0.05). To take the delayed onset of the hemodynamic response into account, the stimulation paradigm was shifted by 2 s. Signal changes were classified as positive if the stimulation signal was larger than the signal of the rest periods. The sum of voxels with positive activation was averaged across stimulation trials. The resulting time courses were normalized to the baseline and linear signal drifts were removed using the function “detrend.”

Time courses of phMRI measurements were determined by summing up the signals of all voxels within the ROI. The resulting time courses were normalized to the baseline and downsampled to a temporal resolution of 4 s. Subsequently, phCBV time courses were exposed to a further correction: Empirically, the MR signal decreased ∼40% during contrast injection. However, the signal drop ($D_{I}$) from each animal deviated slightly from this value. This deviation was corrected by multiplying each CBV signal by the factor $0.4/D_{I}$.

Fluorescence recordings of donor and acceptor signals were normalized to their baseline and downsampled to the temporal resolution of the MR measurements (1 s for fMRI and 4 s for phMRI). FRET time courses with a negative signal drift and functional data with an oscillating baseline were excluded from analysis.

H1 NMR spectra were analyzed using LCModel^50^ and a dedicated simulated basis-set of metabolite spectra based on the density matrix formalism using a spin system simulation software implemented in MATLAB. Relative metabolite concentrations were derived using total Creatine ([Cr+PCr]=8  mmol/L) as an internal reference of concentration. The macromolecule and lipid background signals were parameterized as proposed by Lopez-Kolkovsky et al.^51^ An additional constraint was set to ensure that the background remained stable throughout experiments: per animal only those spectra were examined, for which the total background (sum of all macromolecules and lipids) did not deviate by >10% from the background of the first spectrum in the time series. Spectra exhibiting Cramer–Rao lower bounds >30% for lactate were excluded from analysis.

Spectra were used to calculate the relative increase of cortical lactate level caused by i.v. lactate injection for each animal. Lactate concentrations measured before injection were averaged. A rise in lactate level can be expected during the lactate injection and in the first minutes after the injection.^20^ It was assumed that the maximum lactate concentration of the three spectra recorded during and after injection corresponds to the rise of the lactate level caused by the injection. This maximum lactate concentration was determined for each animal and normalized to the preinjection concentration.

### 2.8 Calculation of FRET-Signals Expected in Twitch-2B-Expressing Animals

Our method for estimating the expected FRET signal of Twitch-2B-expressing animals during sensory stimulation is based on two assumptions: first, the expected FRET signal corresponds to the convolution of the IRF of Twitch-2B and the neuronal response to the applied sensory stimulation. Second, the measured LFP response reflects the neuronal response. Accordingly, the convolution of the measured LFP response and the IRF of Twitch-2B was calculated to determine the expected fFRET signal of Twitch-2B-expressing animals during one cycle of the sensory stimulation [Fig. 1(e)].

For this purpose, the IRF of Twitch-2B was taken from the study of Thestrup et al.^39^ with a temporal resolution of 0.5 s. This time resolution was chosen because it was close to the resolution of the fMRI data (1 s) and the time to peak (ttp) of the IRF of Twitch-2B could still be resolved. The same temporal resolution was used to extract the measured LFP response to sensory stimulation: first, LFP responses to sensory stimulation were averaged across 10 animals. Subsequently, the envelope of the averaged LFP response was obtained with the desired time resolution of 0.5 s. Finally, the convolution of the envelope of the LFP response and the IRF of Twitch-2B was calculated.

### 2.9 Correction of the Hemodynamic Artifact

Ma et al.^38^ have shown that hemodynamic artifacts in the measured fluorescence signal $F_{m}$ can be modeled by an absorption term as shown in Eq. (1). It consists of the true fluorescence signal (F), the change in the absorption coefficients of the excitation ($\Delta \mu_{\mathrm{ex}}$) and the emitted light ($\Delta \mu_{\mathrm{em}}$), as well as the light traveling path length x. For fiber-based recordings, all possible light paths can be combined since the light coming from all emitting cells is measured

$$F_{m}(t)=F(t)\cdot e^{-\sum x(\Delta \mu_{ex}(t)+\Delta \mu_{em}(t))}.$$ (1)

We extended the absorption model by introducing a proportionality constant $a_{1}$ between changes in an absorption coefficient Δμ and changes in the longitudinal relaxations rate $\Delta R_{2}^{*}$

$$\Delta \mu (t)=a_{1}\cdot \Delta R_{2}^{*}(t).$$ (2)

This correlation is justified, considering that the relaxation rate $R_{2}^{*}$ is also dependent on hemodynamics. For small changes in relaxivity, the relative MR signal $S_{r}$ depends on the echo time TE and the change in relaxation rate $\Delta R_{2}^{*}$^52^^,^^53^

$$S_{r}(t)\approx -\mathrm{TE}\cdot \Delta R_{2}^{*}(t).$$ (3)

Summarizing Eqs. (2) and (3) show that changes in an absorption coefficient Δμ are proportional to the relative MR signal $S_{r}$. The absorption coefficient Δμ can be described by a proportionality constant $a_{2}$, which depends on the echo time TE

$$\Delta \mu (t)=-\frac{a_{1}}{\mathrm{TE}}\cdot S_{r}(t)=a_{2}\cdot S_{r}(t).$$ (4)

Combining all fixed constants ($\sum x,a_{2,\mathrm{ex}}$, $a_{2,\mathrm{em}}$) of Eq. (1) into one absorption parameter b, yields the following equation:

$$F_{m}(t)=F(t)\cdot e^{-b\cdot S_{r}(t)}.$$ (5)

In FRET measurements, this equation applies to the signals of donor and acceptor, each with its own proportionality constant ($b_{d}$, $b_{a}$).

#### 2.9.1 Fitting procedure

**Fig. 2:**
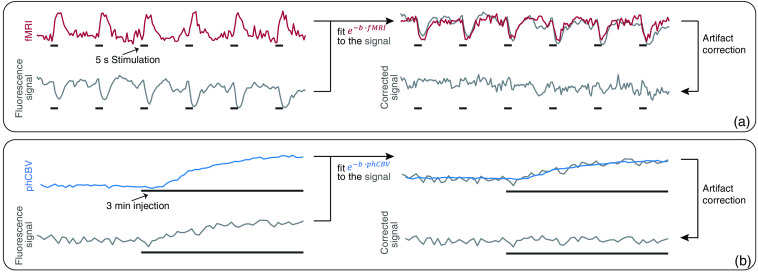
Principle of MRI-based correction method: to each measured fluorescence time course an absorption term was fitted via optimizing the absorption parameter b. For fitting fFRET data, the simultaneously measured fMRI signal was used. For phFRET recordings, the averaged CBV signal was used. Correction of the artifact was performed by dividing the fluorescence signal by the resulting absorption term. Correction procedure for (a) an exemplary fFRET fluorescence and (b) an exemplary phFRET time course.

To determine the proportionality constants, the absorption term $e^{-b\cdot S_{r}(t)}$ was fitted to the measured fluorescence signals by adjusting the absorption parameter b. For fitting of fFRET time courses, the simultaneously recorded fMRI signal was inserted as MR signal $S_{r}$ [Fig. 2(a)]. For fitting of phFRET-signals, time courses of the averaged CBV signals were used [Fig. 2(b)].

The fit was optimized by minimizing the sum of squared residuals using the function “fmincon.” The optimization procedure was repeated for 100 starting points to find the global minimum. Starting points were generated with the function “multistart.” The fitting procedure showed the best results when omitting time points with the baseline signal. Therefore, for fFRET, only active time points were considered in the calculation of the least-squares deviation (stimulation start until 2 s after the end of stimulation). For phFRET, only time points during injections were used.

In the first step, the fluorescence signal of the acceptor fluorophore was fitted. Limits for the parameter $b_{a}$ were set to [−10,10]. Afterward donor signals were fitted. Limits of the parameter $b_{d}$ were set according to the parameter $b_{a}$ (fFRET: $\mathrm{abs}(1.5\times b_{a})\times [-1,1]$; phFRET: $\mathrm{abs}(1.1\times b_{a})\times [-1,1]$). Limiting the parameter $b_{d}$ prevented parts of the actual fluorescence signal were removed by artifact correction.

#### 2.9.2 Artifact correction and FRET-signal calculation

For artifact correction, fluorescence signals were divided by the absorption terms resulting from the fitting procedure. Finally, FRET signals (ratios of uncorrected or corrected fluorescence signals) were calculated according to the specifications of the sensor proteins (Twitch-2B: acceptor signals divided by donor signals,^39^ Laconic: donor signals divided by acceptor signals^18^). FRET signals were reported as percent change from baseline.

For phFRET measurements, the change in FRET signal during injection was determined for each FRET-signal time course (i.e., the difference in FRET signal from injection end and injection start). The first three and the last three data points recorded during each injection were averaged and the difference was calculated. In addition, the signal-to-noise ratio (SNR) was determined for each FRET-signal time course by calculating the ratio of the detected signal change and the standard deviation of the period before injection. Only when the SNR was equal or larger than an arbitrarily chosen threshold (SNR≥3), the detected change was considered to be an actual alteration of the FRET signal.

### 2.10 Data Representation

Throughout the text, results are stated as mean ± standard deviation, or minima and maxima are specified. Figures with time courses show averages and standard deviation. In the case of wide variability of time courses, individual signal courses are shown. If not specified otherwise, individual data clusters are displayed as scatterplots and boxplots. In boxplots the central mark represents the median, boxes include the 25th and 75th percentiles, whiskers extend to the last data points within the 1.5× interquartile, and outliers are indicated as crosses.

Before testing for statistical differences, data were subjected to a Kolmogorov–Smirnov test for normal distribution. Since none of our data were normally distributed, a nonparametric test (U-test) was used for further statistical testing.

## 3 Results

### 3.1 fMRI-FRET

**Fig. 3:**
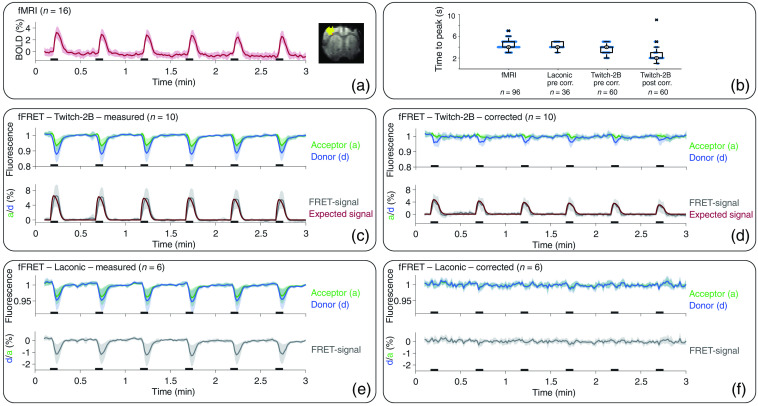
Averaged time courses of fMRI-FRET with standard deviation, exemplary activation map, and ttp: (a) averaged fMRI signal and exemplary activation map. Time courses showed a clear response to each stimulation period (black bars). (b) Overlay of scatter plots and boxplots of ttp’s of fMRI and FRET signals. Note the temporal resolution of 1 s. (c) Time courses of the donor (blue), acceptor (green), and the FRET signal (gray) of Twitch-2B-expressing animals before and (d) after correction. The expected time course of the FRET signal (red) was normalized to the maxima of the actual FRET signal. (e) Fluorescence and FRET-signal time courses of Laconic-expressing animals before and (f) after correction of hemodynamic artifacts.

To assess whether fiber-based *in vivo* detection of calcium and lactate levels is feasible, we performed simultaneous fMRI-FRET measurements with rats expressing either Twitch-2B or Laconic as sensors, respectively. We performed functional measurements with electrical paw stimulation. fMRI activation maps and time courses (n=16) showed a distinct response to stimulation with a ttp of (4.6±1.0)  s and an onset around 1 s after the start of each stimulation train [Figs. 3(a) and 3(b)]. Fluorescence time courses (n=10) of Twitch-2B-expressing animals showed signal changes starting directly with stimulation onset [Fig. 3(c)]. Acceptor signals increased while donor signals decreased. After ∼1  s, a signal decrease of both fluorophores was observed. FRET signals of uncorrected fluorescence data started to rise directly with stimulation onset and peaked at ttp of (3.8±0.9)  s [Fig. 3(b)]. For artifact correction, the absorption term $e^{-b\cdot S_{r}}$ was fitted to the fluorescence signals. For each measurement, the simultaneously recorded fMRI signal was inserted for $S_{r}$. Application of this correction removed major components of the signal decrease [Fig. 3(d)] and the remaining FRET signal had a ttp of (2.7±1.4)  s [Fig. 3(b)]. To compare these data with the theoretically expected signal in artifact-free fluorescence recordings of Twitch-2B-expressing animals, we calculated a reference by convolving the neuronal response with the known IRF of the Twitch-2B sensor. LFP measurements were used to identify the neuronal response to stimulation. The resulting time course showed a ttp of 2.0 s and started to rise directly with stimulation onset. Comparing this time course to our measured data, it became evident that prior to correction, the measured FRET signal deviated from the expected signal curve [Fig. 3(c)]. After applying the correction, FRET-signal time courses agreed well with the expected time course [Fig. 3(d)].

Fluorescence measurements (n=6) of donor and acceptor fluorophores in Laconic-expressing animals showed a signal decrease starting around 1 s after the start of each stimulation train, resembling the signal decrease observed for Twitch-2B-expressing animals [Fig. 3(e)]. The amplitude of signals from donor fluorophores was (31±11)% larger compared with signals from acceptor fluorophores. Accordingly, a decrease was evident in FRET signals of fluorescence data. Ttp of FRET signals was (4.2±0.6)  s [Fig. 3(b)], similar to ttp observed in fMRI measurements. Application of the MR-based correction algorithm removed the signal drops in fluorescence time courses of Laconic expressing animals [Fig. 3(f)]. The averaged time course of corrected FRET signals showed no pronounced signal change triggered by stimulation. However, a U-test revealed significant differences between stimulation and the first 5 s of rest periods, for the averaged corrected FRET signals (p=0.006, Bonferroni corrected). This difference was not found for the uncorrected data (p=0.090, Bonferroni corrected).

### 3.2 phMRI + phFRET

**Fig. 4:**
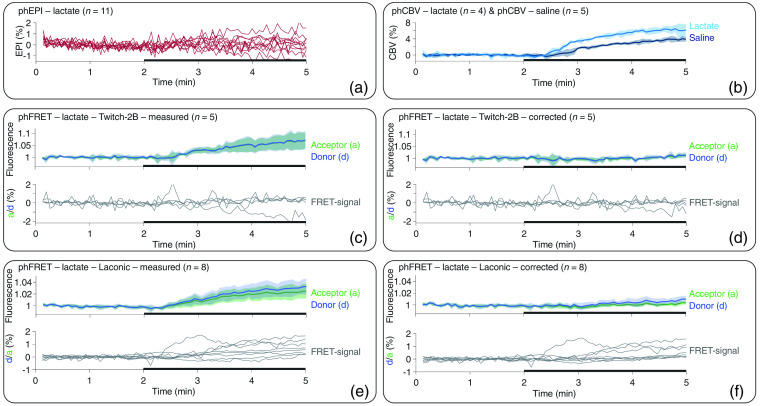
Averaged time courses of phMRI and phFRET measurements for lacate and saline injections. Individual signal time courses are shown for data sets with strongly varying time courses. (a) phEPI signals (red) showed large variations and no clear trend for injection of lactate (black bars). (b) phCBV signal increased during the injection of both lactate (light blue) and saline (dark blue). (c) Time courses of the donor (blue), acceptor (green), and the FRET signal (gray) in Twitch-2B-expressing animals during lactate injection prior to and (d) after artifact correction (right). (e) Fluorescence and FRET-signal time courses of Laconic-expressing animals during lactate injection before and (f) after correction.

To better assess the sensitivity of FRET-based lactate detection, we performed phMRI and phFRET measurements during lactate injection in rats expressing Laconic or Twitch-2B. Signal changes in n=11 phEPI measurements upon lactate injection were low (<1%) and showed large variability, and no clear signal trend was evident [Fig. 4(a)]. Therefore, we deemed the phEPI data unsuitable for MR-based correction of hemodynamic artifacts in fluorescence measurements. In contrast, phCBV measurements showed a substantial signal increase of around 6% and 4% for both lactate (n=4) and saline (n=5) injections, respectively [Fig. 4(b)]. Therefore, phCBV measurements were used for MR-based correction of the fluorescence recordings, and the average of the phCBV signals was inserted into the absorption term $e^{-b\cdot S_{r}}$ for the correction procedure.

**Fig. 5:**
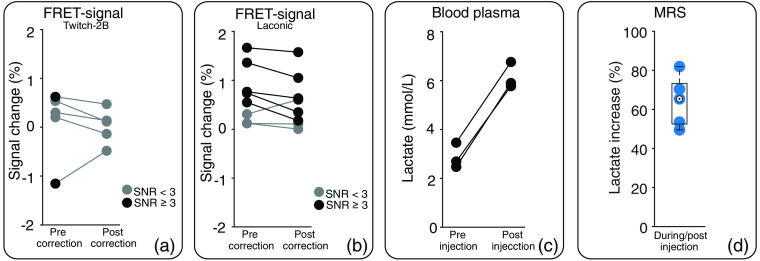
FRET-signal change and increase in lactate level caused by lactate injection. Data points of individual animals are connected by lines. Individual change of the FRET signal in (a) n=5 Twitch-2B-expressing and (b) n=8 Laconic-expressing animals upon lactate injection, before (left) and after (right) application of the correction algorithm. Only values with SNR≥3 (black) were considered as FRET-signal change. Values with SNR<3 (gray) were classified as no signal change. (c) Lactate level in blood plasma before (left) and after injection (right) of lactate (1  mmol/kg) in n=3 animals. (d) Relative increase in cortical lactate level caused by lactate injection (2.5  mmol/kg) as determined by MRS in n=5 animals.

Fluorescence time courses of n=5 Twitch-2B-expressing animals showed a signal increase during lactate injection [Fig. 4(c)], with roughly similar magnitudes for donor and acceptor. Only for two animals, the corresponding FRET signal showed an SNR higher than three [Fig. 5(a)] and the FRET-signal change was considered. After application of the correction, fluorescence signals and FRET signal remained constant during lactate injection [Figs. 4(d) and 5(a)]. In Twitch-2B-expressing animals, fluorescence signals also increased during saline injection (n=2), which was completely removed by the correction (Fig. S3 in the Supplemental Materials).

In Laconic-expressing animals (n=6), prior to correction, both fluorescence signals and FRET-signal time courses showed an increase during lactate injection [Fig. 4(e)]. After correction, the acceptor signal remained stable, whereas the donor signal increased, resulting in a net increase of the FRET signal [Fig. 4(f)]. However, changes in FRET signals showed a large variability between individual Laconic-expressing animals [Fig. 5(b)]. Five-time courses showed an increase in the FRET signal (SNR≥3) before applying the MR-based correction. The signal change was between 0.5% and 1.7%. This increase persisted after correction, albeit with slightly reduced values between 0.2% and 1.6%. One of three-time courses that showed no signal change before correction showed a signal increase of 0.6% after correction, whereas the FRET signals of the other two measurements remained unchanged.

### 3.3 Blood Analysis and H1 MRS

To verify that lactate injection leads to a sufficient increase in lactate levels in blood and brain, blood analysis and H1 MRS experiments were performed, respectively. Blood analysis showed a doubling of the lactate blood plasma level from (2.9±0.5)  mmol/L to (6.1±0.5)  mmol/L, caused by lactate injection [Fig. 5(c)]. One outlier was found in preinjection values of the H1 MRS data using the interquartile range method. Spectra of this animal were excluded from further analysis. Cortical lactate level of remaining animals (n=5) increased by (64±13)% during i.v. lactate injection [Fig. 5(d)].

### 3.4 Absorption Parameters

**Fig. 6:**
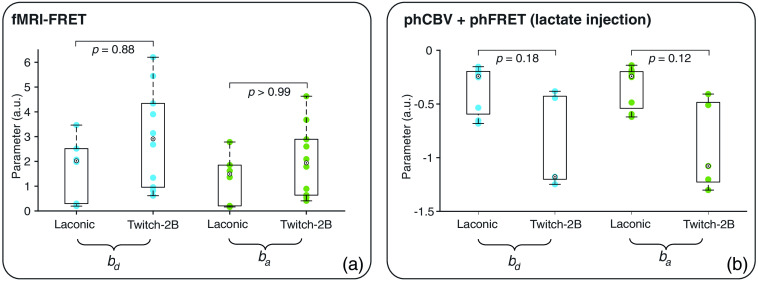
Absorption parameters $b_{d}$ and $b_{a}$ for signals of donor (blue) and acceptor (green) fluorophores for (a) fMRI-FRET and (b) phCBV and phFRET measurements during lactate injection. A U-test was used to test for significant differences.

Absorption parameters $b_{d}$ and $b_{a}$ were calculated by fitting the absorption term $e^{-b\cdot S_{r}}$ to the measured fluorescence separately for donor and acceptor signals, respectively. The calculated parameters ranged from 0.2 to 6.2 for fMRI-FRET measurements [Fig. 6(a)] and from −1.3 to −0.1 [Fig. 6(b)] for phCBV and phFRET measurements with lactate injection. Overall, the absorption parameters showed a large variability with standard deviations ranging from 0.5 to 0.8. Although not significant (U-test, p>0.05, Bonferroni corrected), absolute parameters for measurements with Twitch-2B-expressing animals tended to be larger than those from Laconic-expressing animals. We, therefore, conclude that the absorption strength also depends on the used FRET sensor.

## 4 Discussion

In this study, we performed fiber-based lactate and calcium recordings in combination with fMRI and CBV measurements. Fluorescence signals were measured in the cortex of rats expressing the genetically encoded FRET sensors Laconic and Twitch-2B. When comparing time courses of fluorescence, fMRI, and CBV signals, recorded upon forepaw stimulation and i.v. injections of lactate or saline, we identified prominent effects of hemodynamic artifacts. Artifacts were also present in the ratios of fluorescence signals that corresponded to the calcium and the lactate signals for Twitch-2B and Laconic, respectively. The application of an MR parameter-based correction algorithm yielded results that were consistent with expected signal changes based on LFP recordings, MRS, and blood analysis.

### 4.1 Performance of the MR-Based Correction Algorithm

*In vivo* calcium recordings using Twitch-2B verified that fiber-based FRET measurements are possible and showed that hemodynamic artifact correction is necessary. FRET-based calcium measurements are well suited to examine hemodynamic artifacts. Cortical calcium dynamics are well-understood and closely linked to neuronal activity.^25^^,^^26^^,^^54^^,^^55^ Since the IRF of Twitch-2B is known,^39^ we were able to calculate the expected calcium response to electrical paw stimulation using LFP recordings. Regarding phFRET recordings, we did not expect any calcium release-eliciting effect for saline or lactate injections. Calcium signals of uncorrected FRET data showed a significant deviation from the expected signals for both functional and pharmacological measurements. The high agreement of the corrected FRET signals with expected signals demonstrated the suitability of the presented MR-based correction method.

### 4.2 Fiber-Based Lactate Recordings are Feasible *In Vivo*

We detected changes in lactate level with fiber-based FRET measurements using Laconic, for both sensory stimulation and lactate injections, when the correction algorithm was applied. Uncorrected fluorescence signals clearly matched the BOLD fMRI time courses, which was particularly evident from similar values of onset and ttp. We, therefore, conclude that mainly a hemodynamic artifact was detected. Using the correction algorithm these artifacts were successfully corrected, revealing a small but significant change in lactate signal upon electrical paw stimulation. Previously, lactate changes during electrical brain stimulation or brain needle prick were investigated using microdialysis. Caesar et al.^12^ reported that a change in lactate level was detectable only after 30-s stimulation. In contrast, other studies showed that a shorter duration of stimulation was sufficient to cause a slow lactate rise lasting several minutes, and starting 5 s to 1 min after the stimulation onset.^11^^,^^13^ We did not detect a further lactate increase during resting periods. Hu and Wilson^11^ additionally reported an initial dip in the lactate level that started immediately with stimulation. This initial dip coincides with the lactate change detected in our study.

**Fig. 7:**
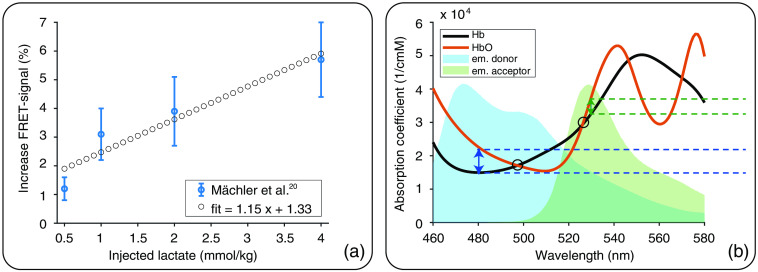
(a) Mächler et al.^20^ showed the increase in FRET signal for Laconic-expressing mice (blue points, average ± standard deviation). According to a linear fit (dotted line), an increase in FRET signal of around 4% can be expected for injection of 2.5 mmol/kg lactate. (b) Absorption coefficients of Hb (black) and HbO (red) are wavelength-dependent.^58^ For the blue and green light emitted by the donor (blue double-headed arrow and dashed lines) and acceptor (green double-headed arrow and dashed lines) fluorophores of Twitch-2B and Laconic, the absorption coefficient of HbO is greater than that of Hb. At the isosbestic points 479 and 526 nm (circles) absorption coefficients of Hb and HbO are identical. Each point lies within the emission spectra of one of the fluorophores^44^^,^^46^ (blue and green background; arbitrary units). If narrow bandpass filters were used, centered at the isosbestic wavelengths, oxygenation change-related artifacts could be minimized. Full absorption and emission spectra of Twitch-2B and Laconic are given in Figs. S2(a) and S2(b) in the Supplemental Materials.

Furthermore, our fiber-based measurements demonstrated a lactate increase of up to 1.6% after i.v. injection. The rise in lactate level was verified by MRS and blood analysis. MRS measurements showed that the cortical lactate level increased around 60% to 70% during injection of lactate. Based on blood analysis, it was estimated that the blood lactate level was increased fivefold after injecting the lactate volume used for MRS (2.5  mmol/kg). However, blood vessels represent only around 2% to 3% of the cortical tissue.^56^^,^^57^ Accordingly, only 15% to 20% of the lactate increase detected by MRS can originate from the increase in blood lactate level in vessels. We, therefore, attribute part of the detected signal change during lactate injection to an increase in intra- and extracellular lactate levels. Histological validation found sensor expression exclusively in neurons, suggesting that signal changes represented an increase in neuronal lactate level. Mächler et al.^20^ found an increase in neuronal lactate level upon i.v. lactate injection in mice using two-photon microscopy. According to that study, an increase in neuronal lactate level of around 4% can be expected for i.v. injection of 2.5  mmol/kg lactate [Fig. 7(a)]. However, a substantially lower signal is expected for measurements using fiber-based detection since it is not possible to examine the signal from individual cells. Instead, a bulk signal is detected from all FRET sensor-expressing cells as well as background signal from the neuropil and auto-fluorescence from tissue.

### 4.3 Hemodynamic Artifacts in FRET Recordings

Hemodynamic changes lead to artifacts in fluorescence recordings since hemoglobin in blood absorbs light. Furthermore, hemodynamic changes modulate MR contrast. However, the generic term “hemodynamics changes” comprises various effects such as changes in blood oxygenation, changes in blood volume as well as blood dilution. Here, we observed that in functional recordings, the effect of oxygenation dominated hemodynamic artifacts (Sec. 4.3.1), whereas in pharmacological measurements oxygenation remained constant and the combination of dilution and volume change was prominent (Sec. 4.3.2).

#### 4.3.1 Oxygenation affects fFRET

We observed hemodynamic artifacts in fFRET measurements and identified changes in oxygenation caused by neuronal activity as their major source. This notion is supported by the following observations: A hemodynamic contribution to the response to sensory stimulation was indicated by the observation of similar values of ttp of fMRI- and FRET signals when uncorrected fluorescence signals were analyzed. Oxygenation changes as the origin of the artifact were concluded from both the positive sign of the absorption parameter b as well as from the larger artifact amplitudes in donor signals as compared with acceptor signals, as explained in the following.

During fMRI measurements, the change in blood oxygenation has a strong effect on contrast, which is known as the BOLD effect.^59^^,^^60^ Our fMRI measurements showed typical BOLD time courses. Onset and ttp corresponded to values expected according to a cortical hemodynamic response function optimized for rats.^49^ Changes in blood oxygenation also affect the detected fluorescence signal, due to different absorption coefficients of oxygenated (HbO) and deoxygenated hemoglobin (Hb). For the FRET sensors used in this study, the absorption coefficients are larger for HbO than for Hb.^58^ Thus, an increase in blood oxygenation will elicit a decrease in fluorescence signals and an increase in the MR signal. Consequently, the absorbance parameter b of the presented absorbance term has a positive sign when deoxygenation of the blood occurs.

To examine this artifact in more detail, we compared the fluorescence spectra of the FRET sensors [Figs. S2(a) and S2(b) in the Supplemental Materials] with the absorption coefficients of HbO and Hb [Fig 7(b)]. This comparison demonstrated that the difference in absorption coefficients between HbO and Hb is twice as large for the emission of the donor fluorophore (around 480 nm) compared with the acceptor fluorophore (around 530 nm). Thus, a larger artifact in the donor indicates a change of oxygenation. Due to the different amplitudes in signals of donor and acceptor fluorophores, the artifact cannot be eliminated by calculating the FRET ratio of the uncorrected fluorescence data. However, the artifact may be minimized by measuring fluorescence at two isosbestic points of Hb and HbO. The two isosbestic points at 479 and 526 nm lie within the emission spectra of donor and acceptor fluorophores, respectively [Fig. 7(b)]. Using narrow bandpass filters centered at these wavelengths might render signal correction unnecessary. However, the remaining fluorescence signal may be too low for fiber-based FRET recordings.

#### 4.3.2 Dilution and blood volume changes affect phFRET

The observed hemodynamic artifacts in fiber-based phFRET recordings were attributed to dilution effects and cerebral blood volume changes, and not to oxygenation changes. Both a decrease in blood volume and blood dilution lead to a decrease in hemoglobin in the ROI and are, therefore, expected to cause a decrease in absorption of fluorescence signals. It is important to note that this absorption change is independent of the wavelength since the ratio of Hb and HbO remains constant, and the resulting hemodynamic artifact is equal in donor and acceptor signals. CBV measurements are similarly affected by a decrease in blood volume and blood dilution, as both are associated with a local decrease of iron nanoparticles. Accordingly, blood dilution and decreasing blood volume lead to an increase in both CBV and fluorescence signals. Consequently, the absorbance parameter b has a negative sign for effects resulting from blood volume changes or dilution.

In our phFRET measurements using Twitch-2B, amplitudes of donor and acceptor signals were equal during the injections, and absorption parameters b had negative signs. We assume that these phFRET measurements represented the pure hemodynamic artifact since we expected no change in calcium level during either lactate or saline injections. We, therefore, conclude that hemodynamic artifacts in phFRET recordings were induced by dilution effects and blood volume changes and oxygenation changes could be excluded as an artifact source. This result was of practical relevance for the analysis of MRS data. Due to the constant oxygenation, no correction of BOLD-induced line-narrowing^10^^,^^61^^,^^62^ was necessary.

While it can be reasonably assumed that i.v. injection of any liquid into the blood causes similar dilution effects, our phCBV measurements showed a higher signal increase during lactate injection than during saline injection. This observation suggests that injection of lactate leads to an additional decrease in blood volume. This notion is supported by evidence that a rise in lactate during physical exercise causes a decrease in blood volume^63^ and that replacing some blood with Ringer’s lactate solution causes a significant decrease in blood volume.^64^

### 4.4 Artifact Correction Is Necessary

We found large variations in absorption parameters and signal amplitude of lactate between animals. These variations may originate from the fiber location relative to pial and penetrating vessels within the cortex^65^^,^^66^: if the implanted fiber is close to the surface of the cortex or one of the diving vessels, the hemodynamic artifact will be larger than for a fiber remote from the closest blood vessel. Furthermore, our results show that the strength of the absorption also depends on the FRET sensors used. Therefore, artifact correction is required separately for each animal and no general correction function can be calculated.

Confocal fluorescence imaging often uses reflectance measurements to correct the fluorescence signal.^23^^,^^37^^,^^38^ However, reflectance and fluorescence light need to have the same wavelength for effective correction. Therefore, this method is not applicable to fiber-based recordings since a bulk signal is measured, making it impossible to distinguish between reflectance and fluorescence light. A recent preprinted study presented a correction method for fiber-based recordings, where fluorescent signals from several fluorescence dyes were recorded simultaneously using fiber and a spectrometer.^33^ However, fiber-based detection usually does not employ spectrometers, requiring substantial modifications to perform corrections based on reflectance measurements. The MR-based method presented in this study provides a feasible alternative, for combined fluorescence-MRI measurements.^25^^–^^32^^,^^34^ It is also readily applicable with separate MRI measurements, as shown here for phCBV. Furthermore, our correction method can be applied to recordings of any fluorescence sensor. A potential limitation of our proposed method is the required downsampling of fluorescence data to the temporal resolution of MR scans, which may cause a loss of kinetic information. However, this was not the case for our study since the fluorescence signals had slow dynamics in all experiments. The half decay time of Twitch-2B is 2.8 s.^67^ Further, the response of cerebral lactate to electrical stimulation is on the timescale of seconds,^14^^,^^15^ and finally, a very slow but constant signal change is expected for phFRET upon a 3-min injection. For other fluorescence recordings with fast kinetics (e.g., calcium imaging with GCaMP6f^68^), line scanning MRI could be used for correction, enabling temporal resolutions of 50 ms.^69^^,^^70^

Interestingly, hemodynamic artifacts are often not reported in fiber-based studies, although their impact on fluorescence measurements has already been demonstrated in the 1990s.^71^ Their magnitude and importance may vary with the choice of detection wavelength and amplitude of fluorescence signal relative to the artifact. Our measurements clearly show that the absorption properties of blood lead to prominent hemodynamic artifacts in fiber-based FRET measurements and require proper correction.

## 5 Conclusions

Fiber-based fluorescence recordings with genetically encoded FRET-based lactate and calcium sensors are possible in the rodent brain. Hemodynamic artifacts are present in both functional and pharmacological FRET measurements and must be corrected for each animal individually. We introduced an MR-based correction algorithm that effectively removes these artifacts for both simultaneous and separate fluorescence-MRI measurements.

## Code, Data, and Material Availability

All data and codes used in this study are available upon direct request.
