# Supplementary material for: Fiber-based lactate recordings with fluorescence resonance energy transfer sensors by applying an magnetic resonance-informed correction of hemodynamic artifacts
Source: Neurophotonics. 2022 May 9;9(3):032212. doi: 10.1117/1.NPh.9.3.032212 (PMC9084224; doi:10.1117/1.NPh.9.3.032212)
Supplement: Supplementary file 1 [file NPh_009_032212_SD001.pdf]

Fiber-based lactate recordings with fluorescence resonance energy transfer (FRET) sensors by applying an MR-informed correction of hemodynamic artifacts

# - Supplemental Materials –

Henriette Lambers[a], Lydia Wachsmuth[a], Dominik Thomas[a], Fawzi Boumezbeur[b], Vanessa Hoesker[a], Bruno Pradier[a], Cornelius Faber[a,*]

[a] University Hospital Münster, Translational Research Imaging Center (TRIC), Clinic for Radiology, Münster, Germany
[b] NeuroSpin, CEA, CNRS, Paris-Saclay University, Gif-Sur-Yvette, France

Suppl. Table 1: Overview of animals and experiments.

experiment	animal #	anaes-thesia	venti-lated	parlay-zed	FRET sensor	injection	injected volume	remarks
fMRI-FRET[a]	1	m[l]	no	no	Laconic	/	/	2 measurements
fMRI-FRET[a]	2	m[l]	no	no	Laconic	/	/	excluded (oscillatory)
fMRI-FRET[a]	3	m[l]	yes	no	Laconic	/	/	/
fMRI-FRET[a]	4	m[l]	yes	yes	Laconic	/	/	/
fMRI-FRET[a]	5	m[l]	yes	no	Laconic	/	/	2 measurements
fMRI-FRET[a]	6	m[l]	no	no	Twitch-2B	/	/	/
fMRI-FRET[a]	7	m[l]	no	no	Twitch-2B	/	/	/
fMRI-FRET[a]	8	m[l]	yes	yes	Twitch-2B	/	/	2 measurements
fMRI-FRET[a]	9-11[j]	m[l]	yes	no	Twitch-2B	/	/	2 measurements
LFP[b]	12-16	m[l]	no	no	/	/	/	/
LFP[b]	17-21	m[l]	no	no	/	/	/	low temp./RR[o]
phEPI-FRET[c]	3-4[h]	m[l]	yes	no	Laconic	lactate	2.5 mmol/kg	phFRET excluded (signal drift)
phEPI-FRET[c]	5[h],22	m[l]	yes	no	Laconic	lactate	2.5 mmol/kg	/
phEPI-FRET[c]	9[h]	m[l]	yes	no	Twitch-2B	lactate	2.5 mmol/kg	/
phEPI-FRET[c]	10[h],11[h],23[j],24[j]	m+i[m]	yes	no	Twitch-2B	lactate	2.5 mmol/kg	/
phEPI[d]	25	m[l]	yes	no	/	lactate	2.5 mmol/kg	/
phEPI[d]	26	m[l]	yes	yes	/	lactate	2.5 mmol/kg	/
phFRET[e]	27	m[l]	no	no	Laconic	lactate	2.6 mmol/kg	/
phFRET[e]	28,29	m[l]	no	no	Laconic	lactate	2.5 mmol/kg	/
phFRET[e]	30	m[l]	no	no	Laconic	lactate	2.6 mmol/kg	low temp./RR[o]
phFRET[e]	31[j], 32[k]	m+i[m]	yes	no	Laconic	lactate	2.5 mmol/kg	/
phFRET[e]	8[h]	m[l]	yes	yes	Twitch-2B	saline	5 ml/kg	excluded (signal drift)
phFRET[e]	33, 34	m[l]	yes	no	Twitch-2B	saline	5 ml/kg	/
blood analysis	35-37[i]	i[n]	no	no	/	lactate	1.0 mmol/kg	/
MRS[f]	11[j],23[j],24[j],38[i]	m[l]	no	no	/	lactate	2.5 mmol/kg	/
MRS[f]	39	m+i[m]	no	no	/	lactate	2.5 mmol/kg	excluded (high baseline)
MRS[f]	31	m+i[m]	no	no	/	lactate	2.5 mmol/kg	low temp./RR[o]
phCBV[g]	32[i],38,40,41	m[l]	no	no	/	lactate	2.5 mmol/kg	/
phCBV[h]	35-37[h],42,43	m[l]	no	no	/	saline	5 ml/kg	/

[a] simultaneous functional MRI and fluorescence resonance energy transfer measurements (fMRI-FRET)
[b] local field potential (LFP)
[c] simultaneous pharmacological echo planar imaging and fluorescence resonance energy transfer measurements (phEPI-FRET)
[d] pharmacological echo planar imaging (phEPI)
[e] pharmacological fluorescence resonance energy transfer measurements (phFRET)
[f] magnetic resonance spectroscopy (MRS)
[g] pharmacological cerebral blood volume measurements (phCBV)
[h] If both fFRET and phFRET were performed in the same animal, measurements were performed successively without delay.
[i] If blood analysis, MRS and/or phCBV were performed in the same animal, measurements were conducted on different days with at least one week between each experiment.
[j] If MRS and FRET measurements were performed in the same animal, MRS was performed first, followed by virus injection at least one week later; finally FRET experiments were performed at least four weeks after virus injection.
[k] If phCBV and FRET measurements were performed in the same animal, phCBV was performed first, followed by virus injection at least six week later; finally FRET experiments were performed at least four weeks after virus injection.
[l] medetomidine (m)
[m] medetomidine + 0.4 % isoflurane (m+i)
[n] 1.8 % isoflurane (i)
[o] temperature below 36.2 °C and/or RR below 35 beats per minutes

Suppl. Table 2: Overview of virus constructs used.

shortcut	virus constructs	serotype	promotor	animals
Laconic (1)[a]	ssAAV-1/2-hSyn1-Laconic-WPRE-hGHp(A)	1	hSyn1	5,12,27,28,31,32
Laconic (2)[a]	ssAAV-9/2-hCMV-dlox-Laconic(rev)-chI(rev)-dlox-WPRE-hGHp(A) +<br>ssAAV-9/2-mCaMKIIα-iCre-WPRE-hGHp(A)	9<br>9	hCMV<br>mCaMKIIα	2,29
Laconic (3)[a]	ssAAV-9/2-hCMV-dlox-Laconic(rev)-chI(rev)-dlox-WPRE-hGHp(A) +<br>ssAAV-9/2-hGFAP-hHBbI/E-iCre-WPRE-bGHp(A)	9<br>9	hCMV<br>hGFAP	1,3
Laconic (4)[a]	ssAAV-9/2-hCMV-dlox-Laconic(rev)-chI(rev)-dlox-WPRE-hGHp(A) +<br>ssAAV-9/2-hGFAP(2.2)-iCre-WPRE-bGHp(A)	9<br>9	hCMV<br>hGFAP(2.2)	3,4
Twitch-2B[b]	pAAV.hSyn1.Twitch2B.WPRE.SV40	1	hSyn1	6-11,23,24

[a] Laconic encoding virus constructs were obtained from the Viral vector Facility at ETH Zurich.
[b] The Twitch-2B encoding virus construct was obtained from Penn Vector Core at University of Pennsylvania.

Suppl. Table 3: Detailed information about components of the fiber-based FRET setup.

acronym	description	company	item #	central wavelength	bandwidth	reflection band	transmission band	cut-on wavelength
458	laser	Coherent, Germany	OBIS LX	/	/	/	/	/
ND	2 neutral density filters	Thorlabs, Germany	NDC-25C-4 + NE506B	/	/	/	/	/
DM 480	dichroic mirror	Thorlabs, Germany	MD480	480	/	415 - 470	490 - 720	/
collimator	fiber collimator	Thorlabs, Germany	PAF2S-11A	/	/	/	/	/
notch	notch filter	Chroma, USA	ZET457NF	457	21	/	/	/
DM 498	dichroic mirror	Thorlabs, Germany	MD498	498	/	452 - 490	505-800	/
BP 479	bandpass filter	Thorlabs, Germany	MF479-40	479	40	/	/	/
LP 480	longpass filter	Edmund Optics, Germany	Hoya Y48	/	/	/	/	480
lens	focused fluorescence light to the detectors	Thorlabs, Germany	LB1471	/	/	/	/	/
D_d	APD (detected light emitted from the donor fluorophore)	Thorlabs, Germany	APD440A2	/	/	/	/	/
D_a	APD (detected light emitted from the acceptor fluorophore)	Thorlabs, Germany	APD440A2	/	/	/	/	/

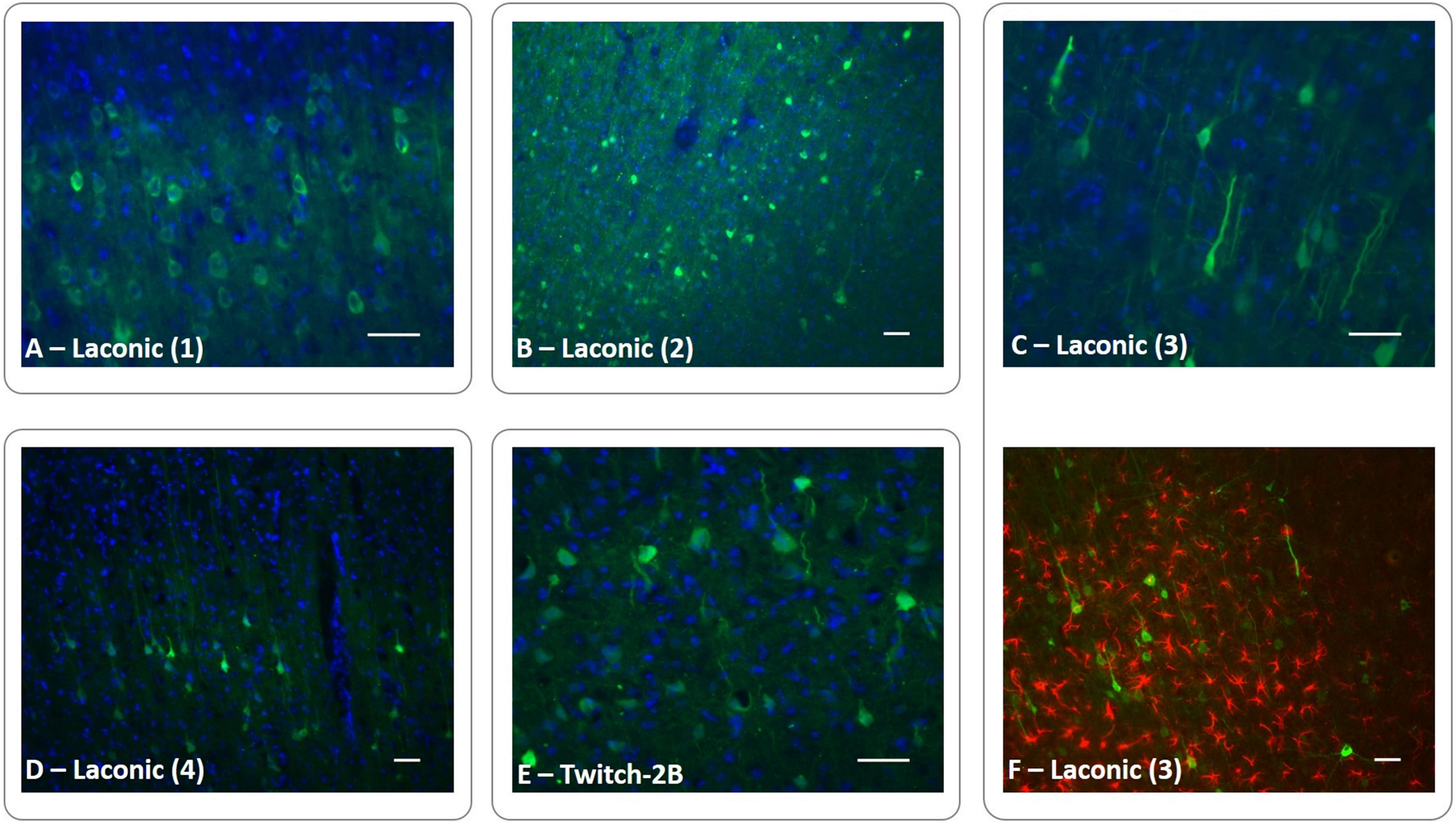

Suppl. Fig. 1: Exemplary histology of S1FL, showing expression of sensor proteins after injection of the virus constructs (Laconic (1-4), Twitch-2B). Virus constructs are listed in detail in Suppl. Table 2. Scale bars indicate 50 µm. (A-E) Overlay of green fluorescent images, showing sensor expression, with blue DAPI co-staining to verify cell viability. (F) Second slice for viral construct Laconic (3), showing overlay of green fluorescent images with red immunostaining for GFAP (astrocytes, mCherry).

All constructs caused FRET sensor expression in neurons. No expression in astrocytes was found, regardless of the promoter (Synapsin, CamKII or GFAP – see Suppl. Table 2) used. Therefore, we did not distinguish between the different viral constructs. Accordingly, we pooled our samples.

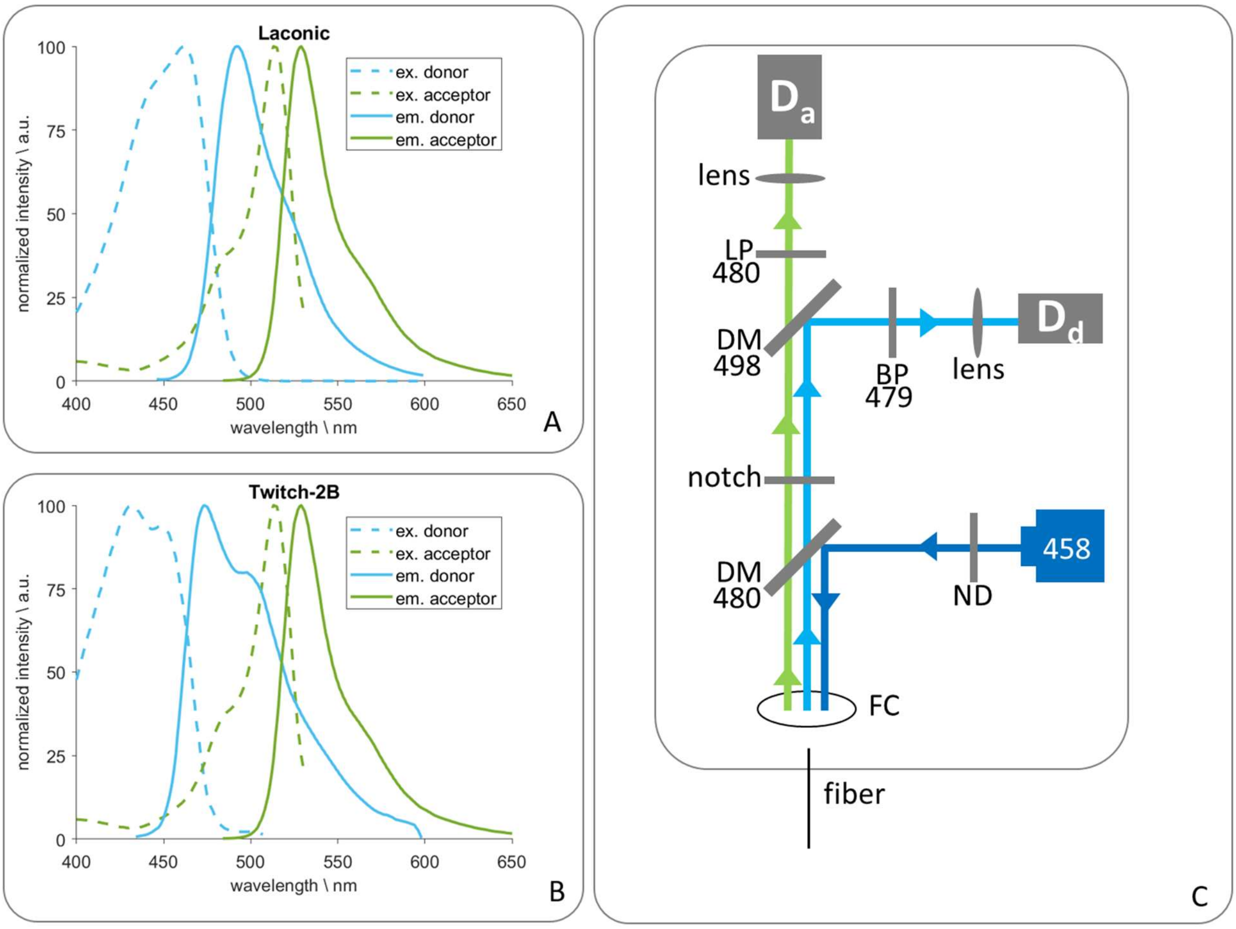

Suppl. Fig. 2: (A) Emission (dashed lines) and absorption spectra (solid lines) of Laconic[18] consisting of mTFP[45] and Venus[46] as donor (blue) and acceptor (green) fluorophore, respectively. (B) Emission and absorption spectra of Twitch-2B[39] consisting of mCerulean3[44] and Venus[46] as donor and acceptor fluorophore, respectively. (C) Detailed schematic of the fiber-based FRET setup: A laser (458 nm) emitted excitation light (dark blue). Excitation light was reduced by neutral density filters (ND), reflected by a dichroic mirror (central wavelength (CWL): 480 nm) and coupled into a fiber. Fluorescent light emitted by the FRET sensors passed the dichroic mirror and a notch filter and was separated using a second dichroic mirror (CWL: 498 nm): Fluorescent light emitted by donor fluorophores (light blue) was reflected, filtered by a bandpass filter (CWL: 479 nm) and focused by a lens to an avalanche photodiode (APD, D_d). Light emitted by acceptor fluorophores (green) passed both dichroic mirrors, a longpass filter (cut-on wavelength: 480 nm) and focused by a lens to a second APD (D_a).

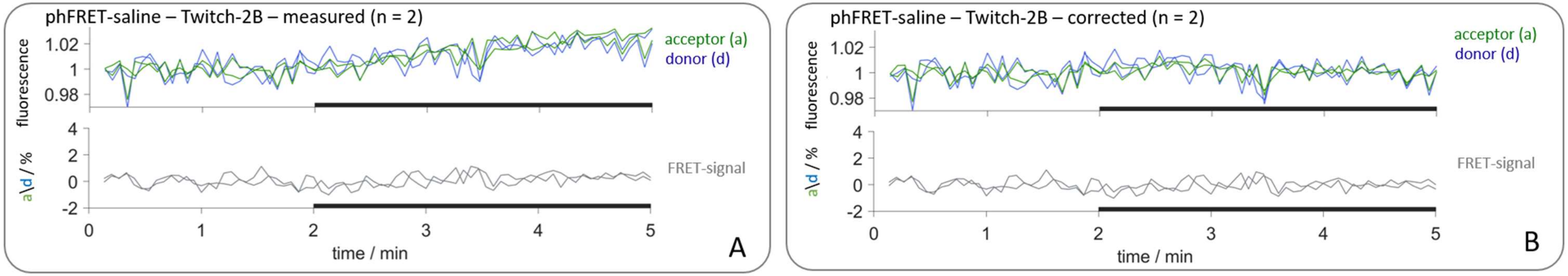

Suppl. Fig. 3: Individual fluorescence and FRET-signal time courses of n = 2 Twicth-2B-expressing animals during saline injection (black bars) (A) before and (B) after applying the correction algorithm.
